# Adherence to exercise in breast cancer survivors during and after active treatment: A systematic review and meta-analysis

**DOI:** 10.1016/j.jsampl.2024.100071

**Published:** 2024-07-12

**Authors:** Martin Ackah, Ioulia Barakou, Ulric Sena Abonie, Florentina Johanna Hettinga

**Affiliations:** a Department of Sport Exercise and Rehabilitation, Northumbria University, Newcastle upon Tyne, NE1 8ST, United Kingdom; b Department of Nursing, Midwifery & Health, Northumbria University, Newcastle upon Tyne, NE7 7XA, United Kingdom

**Keywords:** Physical activity, Exercise oncology, Fatigue, Exercise engagement, Exercise motivation, Well-being

## Abstract

**Background:**

Ensuring adherence to exercise programs is important for optimizing benefits and efficacy of interventions in women with breast cancer. Despite numerous studies on adherence to exercise in women with breast cancer, no systematic review has exclusively examined exercise adherence and its influencers during and after active treatment in this population. This review aims to examine the adherence rates and influencing factors for exercise in breast cancer survivors during and after treatment.

**Methods:**

We systematically searched PubMed, CINAHL, Web of Science, and Scopus. We included studies on adherence to exercise and potential influencing factors conducted on women with breast cancer. Relevant studies were screened, and data were extracted. Analyses of adherence and factors influencing adherence were performed for ‘during’ and ‘after’ primary cancer treatment. Systematic review and meta-analyses were performed.

**Results:**

Twenty-six studies were included. The overall pooled exercise adherence was 64% (95% CI: 58%–70%). Adherence to exercise during primary cancer treatment was 63% (95% CI: 55%–70%), and after primary cancer treatment was 68% (95% CI: 59%–78%), with no significant variation (Q ​= ​0.82, p ​= ​0.36). Physical fitness, baseline physical activity, fatigue, education, body mass index, and having a partner were identified to influence adherence during primary cancer treatments. Body mass index was reported to have a negative association with exercise adherence during and after primary cancer treatment.

**Conclusions:**

The review revealed no significant variations in exercise adherence among women with breast cancer both during and after primary cancer treatments. Body mass index appeared to be negatively associated with both stages of primary cancer treatment.

## 1 Background

Breast cancer remains a prominent global health concern, with an estimated incidence of nearly 12%, surpassing that of lung cancer (11.4%) in 2020 [1]. Recent advances in the clinical management of breast cancer have resulted in increased survival rates over the past decades [2]. Accordingly, there is a growing focus on rehabilitation interventions that enhance overall well-being before, during, and after treatment to improve quality of life of survivors [3,4]. Exercise plays an important role in enhancing the well-being of adults with cancer, including persons with breast cancer [5]. Studies have shown the beneficial impact of exercise programs on various components of health, such as on aerobic fitness, muscle strength, quality of life, fatigue, and depression [[5], [6], [7]]. Current guidelines recommend cancer survivors engage in aerobic or resistance or combined aerobic plus resistance training at least three times per week [5]. In addition, the World Health Organization advises all adults, even those with chronic conditions (e.g. cancer), to participate in 150–300 ​min of moderate-intensity aerobic exercise, 75–150 ​min of vigorous-intensity aerobic exercise, or a mix of both each week for significant health benefits [8]. Despite these recommendations, few women with breast cancers were less likely to meet exercise/physical activity guidelines [[9], [10], [11]].

In the context of evaluating adherence to exercise programs, the absence of a universally accepted gold standard is apparent. As a result, measures such as session attendance [12], adherence to recommended exercises from healthcare providers [13,14], adherence to prescribed exercise thresholds [15], and participants meeting a predefined exercise goal for duration and intensity [16] are commonly used to assess adherence levels in rehabilitation programs. Given the benefits associated with exercise to women with breast cancer, adherence to exercise program is imperative. This is essential not only to optimize the participants' benefits from the program but also to ensure the efficacy of the intervention [14,17,18]. A review of heterogeneous cancer populations reported that adherence to exercise programs varied, with rates of 73% during the pre-treatment phase, 68%–91% during the active treatment phase, and 78% after primary cancer treatment [19].

A systematic review of barriers and facilitators of exercise experienced by cancer survivors identified fatigue, time constraints, insufficient patients’ information, and treatment-related side effects as key barriers to exercise for cancer survivors [20]. Additionally, two reviews highlighted a positive correlation between individuals' exercise history and adherence to exercise regimens [19,21]. These reviews explored heterogenous cancer survivor types. Additionally, there is a suggestion that the factors impacting exercise adherence may vary during and after primary cancer treatment [22]. Indeed, Kampshoff et al. [19] underscored that the limited number of studies impeded their review from examining whether determinants of exercise adherence exhibit variations across cancer types, exercise modalities, modes, and intervention delivery.

The variance in exercise adherence rates thus suggest that in addition to treatment stage, cancer type may influence adherence. However, adherence to exercise among women with breast cancer has not been systematically reported. Hence, addressing this gap is essential to guide treatment efforts. Although numerous studies have been conducted on women with breast cancer, no systematic review and meta-analysis have exclusively focused on adherence and the factors influencing exercise adherence among women with breast cancer during and after active treatment. Therefore, the aim of this review is to explore the rate of adherence and the influencing factors for exercise in women with breast cancer during and after treatment. We hypothesized that there would be a significant difference in exercise adherence estimates between during and after treatment among women with breast cancer.

## 2 Materials and methods

This review was registered prospectively in the International Prospective Register of Systematic Reviews (PROSPERO) database, with the distinctive registration number CRD42024506746. The reporting of this systematic review was conducted following the guidelines outlined by the Preferred Reporting Items for Systematic Reviews and Meta-Analyses (PRISMA) [23].

### 2.1 Inclusion and exclusion criteria

The study aimed to include adult women (18 years and older) living with breast cancer worldwide. All stages of breast cancer were considered. Additionally, the review encompassed breast cancer survivors during and after primary cancer treatment, including chemotherapy, radiation, or adjuvant therapy. Randomized controlled trials and observational studies reporting adherence and factors associated with adherence were included. Only full English papers were considered. However, qualitative studies, systematic reviews, and protocol papers were excluded.

### 2.2 Search strategy for identification of studies

Studies estimating adherence to exercise, and associated factors were searched. The search spanned four databases: PubMed, CINAHL, Web of Science, and Scopus. The search was limited to English language peer-reviewed publications from January 2000 to 9th February 2024. Relevant keywords and MeSH terms included “breast cancer"[All Fields], “breast neoplasms"[MeSH Terms], exercise [MeSH Terms], and “adherence"[All Fields]. The complete search strategy is presented in Table 1. Moreover, manual searches of relevant articles were conducted for additional studies.

**Table 1:** Search strategy for PubMed, adapted for other databases.

Search term
(“breast cancer”[All Fields] OR (“breast neoplasms”[MeSH Terms] OR (“breast”[All Fields] AND “neoplasms”[All Fields]) OR “breast neoplasms”[All Fields] OR (“breast”[All Fields] AND “tumor”[All Fields]) OR “breast tumor”[All Fields]) OR (“breast neoplasms”[MeSH Terms] OR (“breast”[All Fields] AND “neoplasms”[All Fields]) OR “breast neoplasms”[All Fields] OR (“breast”[All Fields] AND “neoplasm”[All Fields]) OR “breast neoplasm”[All Fields]) OR (“breast neoplasms”[MeSH Terms] OR (“breast”[All Fields] AND “neoplasms”[All Fields]) OR “breast neoplasms”[All Fields] OR (“breast”[All Fields] AND “carcinoma”[All Fields]) OR “breast carcinoma”[All Fields])) AND (“exercise”[MeSH Terms] OR “exercise”[All Fields] OR “exercises”[All Fields] OR “exercise therapy”[MeSH Terms] OR (“exercise”[All Fields] AND “therapy”[All Fields]) OR “exercise therapy”[All Fields] OR “exercising”[All Fields] OR “exercise s”[All Fields] OR “exercised”[All Fields] OR “exerciser”[All Fields] OR “exercisers”[All Fields] OR (“exercise”[MeSH Terms] OR “exercise”[All Fields] OR (“physical”[All Fields] AND “activity”[All Fields]) OR “physical activity”[All Fields]) OR ((“physical examination”[MeSH Terms] OR (“physical”[All Fields] AND “examination”[All Fields]) OR “physical examination”[All Fields] OR “physical”[All Fields] OR “physically”[All Fields] OR “physicals”[All Fields]) AND (“education”[MeSH Subheading] OR “education”[All Fields] OR “training”[All Fields] OR “education”[MeSH Terms] OR “train”[All Fields] OR “train s”[All Fields] OR “trained”[All Fields] OR “training s”[All Fields] OR “trainings”[All Fields] OR “trains”[All Fields])) OR (“physical fitness”[MeSH Terms] OR (“physical”[All Fields] AND “fitness”[All Fields]) OR “physical fitness”[All Fields]) OR (“walked”[All Fields] OR “walking”[MeSH Terms] OR “walking”[All Fields] OR “walks”[All Fields]) OR “recreational physical activity”[All Fields] OR “household physical activity”[All Fields]) AND (“predictor∗”[All Fields] OR “factor∗”[All Fields] OR (“influence”[All Fields] OR “influenced”[All Fields] OR “influences”[All Fields] OR “influencing”[All Fields]) OR (“adherance”[All Fields] OR “adhere”[All Fields] OR “adhered”[All Fields] OR “adherence”[All Fields] OR “adherences”[All Fields] OR “adherent”[All Fields] OR “adherents”[All Fields] OR “adherer”[All Fields] OR “adherers”[All Fields] OR “adheres”[All Fields] OR “adhering”[All Fields]) OR (“attend”[All Fields] OR “attendance”[All Fields] OR “attendances”[All Fields] OR “attendant”[All Fields] OR “attendant s”[All Fields] OR “attendants”[All Fields] OR “attended”[All Fields] OR “attendence”[All Fields] OR “attendents”[All Fields] OR “attender”[All Fields] OR “attenders”[All Fields] OR “attending”[All Fields] OR “attendings”[All Fields] OR “attends”[All Fields]))

### 2.3 Screening and data extraction

The results obtained from the databases were transferred to the Mendeley citation manager, where duplicate articles were eliminated. Subsequently, MA and IB independently screened the titles and abstracts. The full texts papers were then screened, retrieved, and assessed by MA, with a secondary examination by IB. Discrepancies were resolved through discussion with MA and IB. Data extraction was performed MA. Extracted information included the first author's name and year of publication, country, study design, age, sample size, definition of adherence, estimated adherence, and factors influencing adherence.

### 2.4 Quality assessment

The quality assessment in this study was adopted from previous studies [19,24]. It involved examining three key domains: the participation of the study population, handling of study attrition, and the procedures for data collection and analysis. Following the approach outlined by Kampshoff and colleagues [19], a positive score was assigned if the study provided information on a quality parameter and met the specified criterion. Conversely, a negative score was given if the study provided information but failed to meet the criteria. When there was a lack of information or insufficient details, the quality item was marked with a question mark. It is imperative to highlight that within the set of eleven questions employed for the assessment, two questions [F ​= ​if adherence was measured with reliable tool and G ​= ​if adherence was measured with valid tool] were excluded from the computation and were marked with a question mark. They were excluded from the computation because the studies did not provide enough information on these measures to judge these questions. Consequently, the potential range for the maximum attainable score in methodological quality ranged from 0 to 9. The classification of a study as possessing ‘high methodological quality’ was contingent upon achieving a score equal to or exceeding 70% of the designated criteria as positive (+), while a designation of ‘low methodological quality’ was assigned to studies scoring below this threshold [19,25].

### 2.5 Data synthesis

Stata version 16 and Microsoft excel 2013 were used to perform all statistical analyses. First, we systematically discussed the adherence during and after treatment of the included studies. Then, a meta-analysis was performed. The ‘Metaprop command’, as well as the Clopper-Pearson method (i.e., ‘cimethod (exact)’), was used to determine the study-specific confidence intervals [26]. Subsequently, effect sizes and standard errors of the effect sizes were then used to meta-set. The adherence to exercise was pooled using a random-effects model. Heterogeneity was quantified using both Cochrane's Q statistic and the I^2^ statistic [27]. Sub-group analysis was employed to investigate potential variations in estimates based on moderators such as the stage of treatment (during vs after primary cancer treatment), study design (randomized controlled trial vs observational study), environment (Centre-based vs home-based), the definition of adherence (meeting a physical activity/exercise guideline/protocol vs attending sessions vs performing prescribed exercises), and duration of intervention (≥12 weeks vs ​> ​12 weeks). The funnel plot and Egger's regression test were used to assess objective and subjective publication bias respectively. Factors influencing adherence to exercise were eligible for inclusion if they had been adjusted, significant, and reported in the included studies. Significance was attributed to a factor if it was reported in at least two studies [28,29]. Furthermore, it is important to emphasize that the factors influencing adherence during and after primary breast cancer treatment were merely summarized from individual studies, with no additional analysis conducted.

## 3 Results

### 3.1 Screening and characteristics of the included studies

**Fig. 1:**
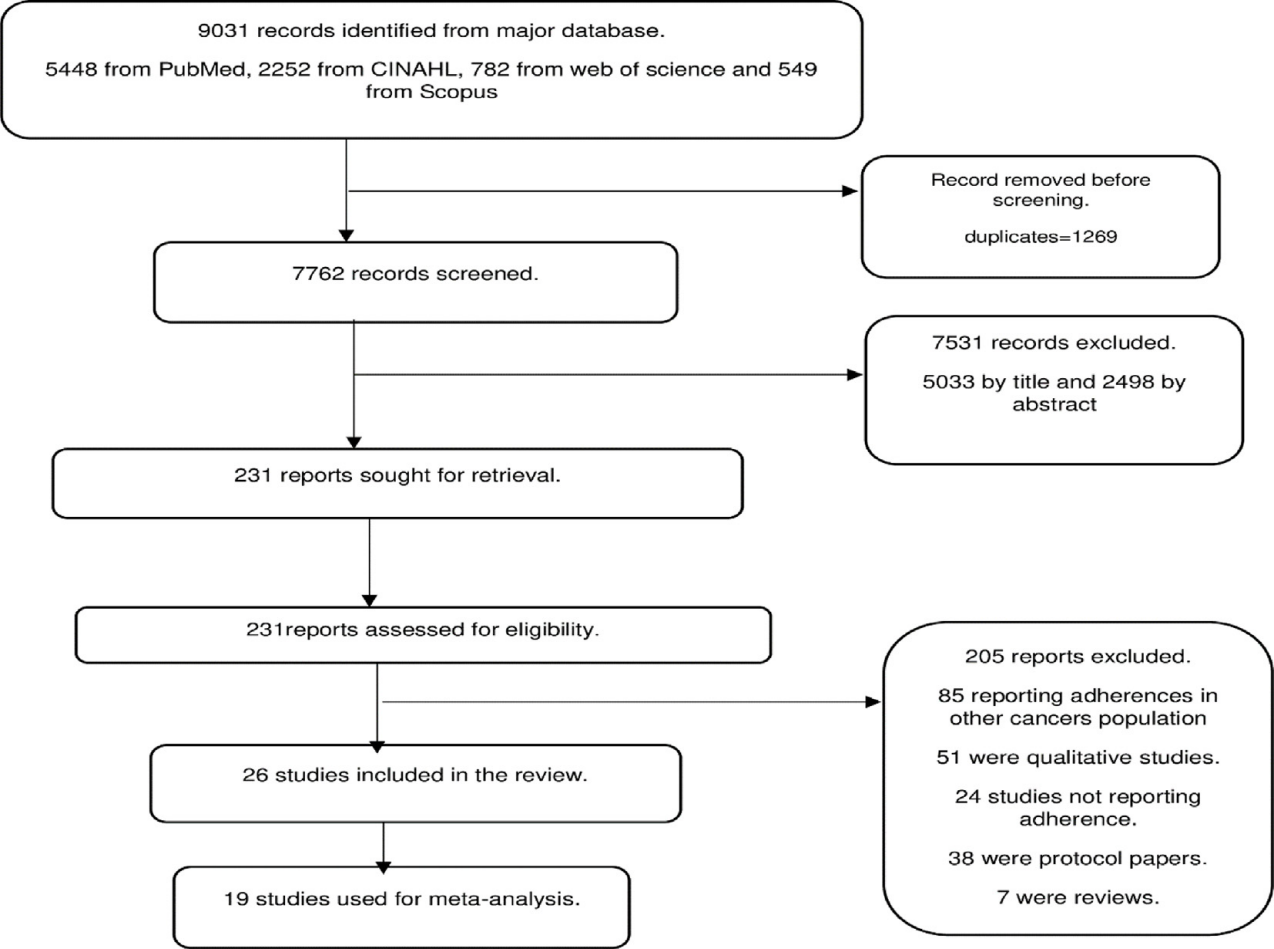
PRISMA flow diagram for screening the articles.

The study identified 9031 records from the major databases: 5448 from PubMed, 2252 from CINAHL, 782 from Web of Science, and 549 from Scopus. After removing 1269 duplicates before screening, 7762 records remained. Following the screening of abstracts and titles, 7531 reports were excluded. 231 reports were assessed for eligibility, with 205 reports subsequently excluded. Common reasons for exclusion included adherence in other cancer populations, qualitative studies, studies not reporting adherence, protocol papers, and systematic reviews. Overall, 26 studies [13,16,[30], [31], [32], [33], [34], [35], [36], [37], [38], [39], [40], [41], [42], [43], [44], [45], [46], [47], [48], [49], [50], [51], [52], [53]] were included (Fig. 1).

A total of 2708 women with breast cancer were included. The studies were published between 2001 and 2024. The majority of the studies were conducted in the USA (n ​= ​11) [13,16,39,41,44,45,[49], [50], [51],^53^] and Canada (n ​= ​5) [30,[33], [34], [35],^40^]. Eighteen studies were randomized controlled trials [16,[31], [32], [33],[35], [36], [37],[42], [43], [44], [45], [46], [47], [48], [49],[51], [52], [53]], and eight were observational studies [13,30,34,36,[38], [39], [40], [41]]. The definition of adherence, adherence rate and factors influencing adherence are reported in Table 2.

**Table 2:** Study characteristics.

Stage	Author	Year	Study design	Location	Age(years) [n ​= ​2708]	Definition of adherence	Adherence rate	Factors influencing adherence
During Treatment	Arem et al. [16]	2016	RCT	Centre/supervised	62 ​± ​7[61]	Meet 150 ​min/wk aerobic goal via daily logs and supervised sessions	72% and 70% strength training session at 6-and-12month respectively and 30% reached the goal of 150 ​mins/wk.	Higher VO2max at baseline, advanced age
	Bland et al. [40]	2018	Observational study	Centre/supervised	51.2 ​± ​10.7[68]	number of exercise sessions attended	64%, and 67% for adjuvant chemotherapy and radiation, respectively	income, employed, higher quality of life
	Bolam et al. [47]	2019	RCT	Centre/supervised	53.4 ​± ​10.1[160]	number of participants achieving nine out ten of the exercise sessions as planned.	68% and 63% for resistance and aerobic, respectively	not reported
	Courneya et al. [33]	2008	RCT	Centre/supervised	25–78[242]	number of sessions attended	70.2%	Gym location/center, VO2max, disease stage, depression
	Courneya et al. [35]	2009	RCT	Centre/supervised	25–78[201]	meeting exercise guideline	36.8% met either aerobic or resistance exercise guideline, and 20.9% met both aerobic and resistance	pretrial exercise, young age, type of surgery, VO2max, BMI, muscle strength, fatigue, instrumental attitude
	Goldschmidt et al. [32]	2024	RCT	Centre/supervised	50.1 ​± ​11.1[122]	Number sessions completed	44.1%	married, education, BMI, patient rating of exercise, nausea
	Hornsby et al. [49]	2014	RCT	Centre/supervised	51 ​± ​6[20]	number of exercise sessions attended, and number of exercise sessions completed	attendance ​= ​82% and adherence ​= ​66%	not reported
	Huang et al. [52]	2015	RCT	Home-based	48.3 ​± ​8[78]	number of prescribed exercises completed	87.1%	fatigue, interest in exercise
	Kirkham et al. [30]	2018	Observational study	Centre/supervised	29–77[68]	number of exercise session attended	58%	not reported
	Lund et al. [31]	2019	RCT	Home-based and supervised	45–74[62]	number of exercise sessions attended and percent of the expected number of sessions.	55% adhered to home base exercise and 65% adhered to supervised exercise	obesity, low muscle strength, neoadjuvant chemotherapy
	Mock et al. [45]	2005	RCT	Home-based	30–69[54]	‘Engaging in ≥60 ​min of aerobic activity weekly for ≥2/3 (67%) of the duration of the trial’	72%	not reported
	Nyrop et al. [13]	2018	Observational study	Home-based	24–64[100]	meeting 44,000 steps/wk. or 6286 steps/day	19%	white race and self-reported walking minutes prior to treatment
	Pickett et al. [44]	2002	RCT	Home-based	31–50[23]	not reported	67%	not reported
	Swenson et al. [39]	2010	Observational study	Home-based	40–55[36]	meeting exercise prescription of 10,000-step protocol per week	67%	Baseline physical inactivity
	van Waart et al. [37]	2020	RCT	Home-based and supervised	50.2 ​± ​9.3[153]	meeting 30mins physical active on at least 75% of prescribed days or attending at least 75% of the prescribed session	56% adhered to home-based exercise and 59% adhered to the supervised sessions	baseline endurance time, higher disease stage, having a partner
	Witlox et al. [36]	2019	RCT	Centre/supervised	50.2 ​± ​7.8[92]	number of sessions attended	83%	education, BMI, and fatigue
	Schmidt et al. [42]	2014	RCT	Centre/supervised	52.7 ​± ​10[95]	number of sessions attended	71%	not reported
	Tao et al. [38]	2020	Observational study	Home-based	18+ [632]	Meeting functional exercise program delivered by medical personnel	68.2%	self-efficacy, social support
After Treatment	Bland et al. [40]	2018	observational study	Centre/supervised	51.2 ​± ​10.7[68]	number of exercise sessions attended	54%	not married, quality of life
	Courneya et al. [34]	2001	Observational study	Centre/supervised	51 ​± ​6.9[24]	number of training sessions attended	66%	Intention
	Daley et al. [48]	2007	RCT	Centre/supervised	30-65[34]	attending at least 70% of the session	77%	not reported
	Latka et al. [51]	2009	RCT	Home-based and supervised	56.5 ​± ​9.5[37]	Meeting 150 ​min/week guideline	81%	lower BMI and higher Stage of Change
	Mathews et al. [50]	2007	RCT	Centre/supervised	51 ​± ​9[24]	number of exercise sessions completed	94%	not reported
	McGuire et al. [53]	2011	RCT	Home-based and supervised	35–75[120]	Number of exercises sessions completed at the end of 24-month study	61.89%	feedback, adherence to previous exercise program
	Milne et al. [46]	2008	RCT	Centre/supervised	36-71[58]	Number of sessions completed	61.3%	not reported
	Pinto et al. [43]	2009	RCT	Home-based	53.4 ​± ​9[43]	Meeting exercise goal	69.76%	baseline self-efficacy for exercise
	Smith et al. [41]	2016	Observational study	Home-based	55.6 ​± ​12.8[193]	meeting physical activity guidelines	54%	Post-diagnosis BMI, surgical treatment

### 3.2 Quality assessment of the included studies

The methodological quality score ranged from 33% to 100%, with a median score of 56%. One study [44] had a low methodological score, while two studies [32,38] achieved a high methodological score.

Seven studies [32,33,35,38,40,41,53] obtained a quality score of ≥70%. Out of all the studies, 42% had concerns with the study sample (item C), 65% had sample size concerns (item D), and 46% and 71%, respectively, had concerns with analytical methodologies (item I and item K). See Table 3.

**Table 3:** Quality assessment of the included studies.

Author	Year	study population and participation.			Study attrition.		Data collection			Data analysis			Total score/9	Quality score (%)
		A	B	C	D	E	F	G	H	I	J	K		
Arem et al. [16]	2016	+	+	+	–	–	?	?	+	+	–	–	5	56
Bland et al. [40]	2018	+	+	+	–	+	?	?	+	+	+	–	7	78
Bolam et al. [47]	2019	+	+	–	+	–	?	?	+	–	–	–	4	44
Courneya et al. [34]	2001	+	+	–	–	–	?	?	+	+	+	–	5	56
Courneya et al. [33]	2008	+	+	–	+	+	?	?	+	+	–	+	7	78
Courneya et al. [35]	2009	+	+	–	+	+	?	?	–	+	+	–	7	78
Daley et al. [48]	2007	+	+	+	–	+	?	?	+	–	–	–	5	56
Goldschmidt et al. [32]	2024	+	+	+	+	+	?	?	+	+	+	+	9	100
Hornsby et al. [49]	2014	+	+	–	–	+	?	?	+	–	–	–	4	44
Huang et al. [52]	2015	+	+	+	–	?	?	?	+	+	+	–	6	67
Kirkham et al. [30]	2018	+	+	+	–	?	?	?	+	–	–	–	4	44
Latka et al. [51]	2009	+	+	+	–	+	?	?	+	–	–	–	5	56
Lund et al. [31]	2019	+	+	+	–	+	?	?	+	–	–	–	5	56
Mathews et al. [50]	2007	+	+	+	–	–	?	?	+	–	–	–	4	44
McGuire et al. [53]	2011	+	+	–	+	+	?	?	+	+	+	+	8	89
Milne et al. [46]	2008	+	+	+	–	–	?	?	+	–	–	–	4	44
Mock et al. [45]	2005	+	+	–	–	?	?	?	+	+	+	–	5	56
Nyrop et al. [13]	2018	+	+	+	+	?	?	?	+	–	–	–	5	56
Pickett et al. [44]	2002	+	+	–	–	?	?	?	+	–	–	–	3	33
Pinto et al. [43]	2009	+	+	–	–	+	?	?	+	+	+	–	6	67
Schmidt et al. [42]	2014	+	+	+	–	?	?	?	+	–	–	–	4	44
Smith et al. [41]	2016	+	+	+	+	?	?	?	+	+	+	+	8	89
Swenson et al. [39]	2010	+	+	–	–	+	?	?	+	+	–	–	5	56
Tao et al. [38]	2020	+	+	+	+	?	?	+	+	+	+	+	9	100
van Waart et al. [37]	2020	+	+	+	+	?	?	?	+	–	–	–	5	56
Witlox et al. [36]	2019	+	+	–	–	?	?	?	+	+	+	–	5	56

A ​= ​Description of cancer type, stage and treatment, B ​= ​Description of inclusion and exclusion criteria, C= Positive if the participation rate at baseline was at least 80%, or if the non-response was not selective, D ​= ​Number of patients included in the analysis ≥100, E ​= ​Positive if the response at first follow-up was at least 80%, or if the non-response at first follow-up was not selective, F= Positive if determinants of adherence were measured with a reliable tool, G ​= ​Positive if determinants of adherence were measured with a valid tool, H ​= ​Adherence was measured by an objective tool, I ​= ​Multivariate analysis techniques was used, J ​= ​Results were presented as point estimates (mean differences/Beta's/correlation coefficients) and measures of variability (SD, standard error or CI), K=Positive if number of samples is at least 10 times the number of independent variables.

### 3.3 Systematic review of exercise adherence during primary breast cancer treatment

Overall, seventeen studies reported 20 adherence definitions [13,16,[30], [31], [32], [33],[35], [36], [37], [38], [39], [40],^42^,^45^,^47^,^49^,^52^]. It is important to highlighted that two adherence definitions were provided each for Hornsby et al. [49], Lund et al. [31], and van Waart et al. [37] during cancer treatment. In eight studies [[30], [31], [32], [33],^36^,^40^,^42^,^49^] exercise adherence was defined as the percentage of sessions attended and ranged from 44% [32] to 83% [36]. In seven studies [13,16,35,[37], [38], [39],^49^] adherence was defined as meeting agreed-upon exercise guidelines and ranged from 19% [13] to 68% [39]. In five other studies [31,37,45,47,52], adherence was defined as meeting the prescribed threshold of exercise regimens and ranged from 59% [37] to 87% [52].

### 3.4 Systematic review of exercise adherence after primary breast cancer treatment

Nine studies [34,40,41,43,46,48,50,51,53] reported on adherence to exercise after cancer treatment. It is worth noting that Bland et al. [40] provided data for both during and after breast cancer treatment. The adherence rates after breast cancer treatment varied between 54% [41] and 94% [40]. Specifically, two studies determined adherence based on attended sessions, and reported rates from 54% [40] to 66% [34]. Additionally, three studies [41,43,51] examined the achievement of exercise goals/guidelines as a measure of adherence, and reported rates ranging from 54% [41] to 81% [51]. Furthermore, three studies [46,50,53] measured the number of completed exercise sessions as a measure of adherence, and revealed rates between 61% [46] and 94% [50]. Lastly, one study defined adherence as meeting the prescribed threshold of exercise regimens [48].

### 3.5 Meta-analysis of exercise adherence in women with breast cancer

**Fig. 2:**
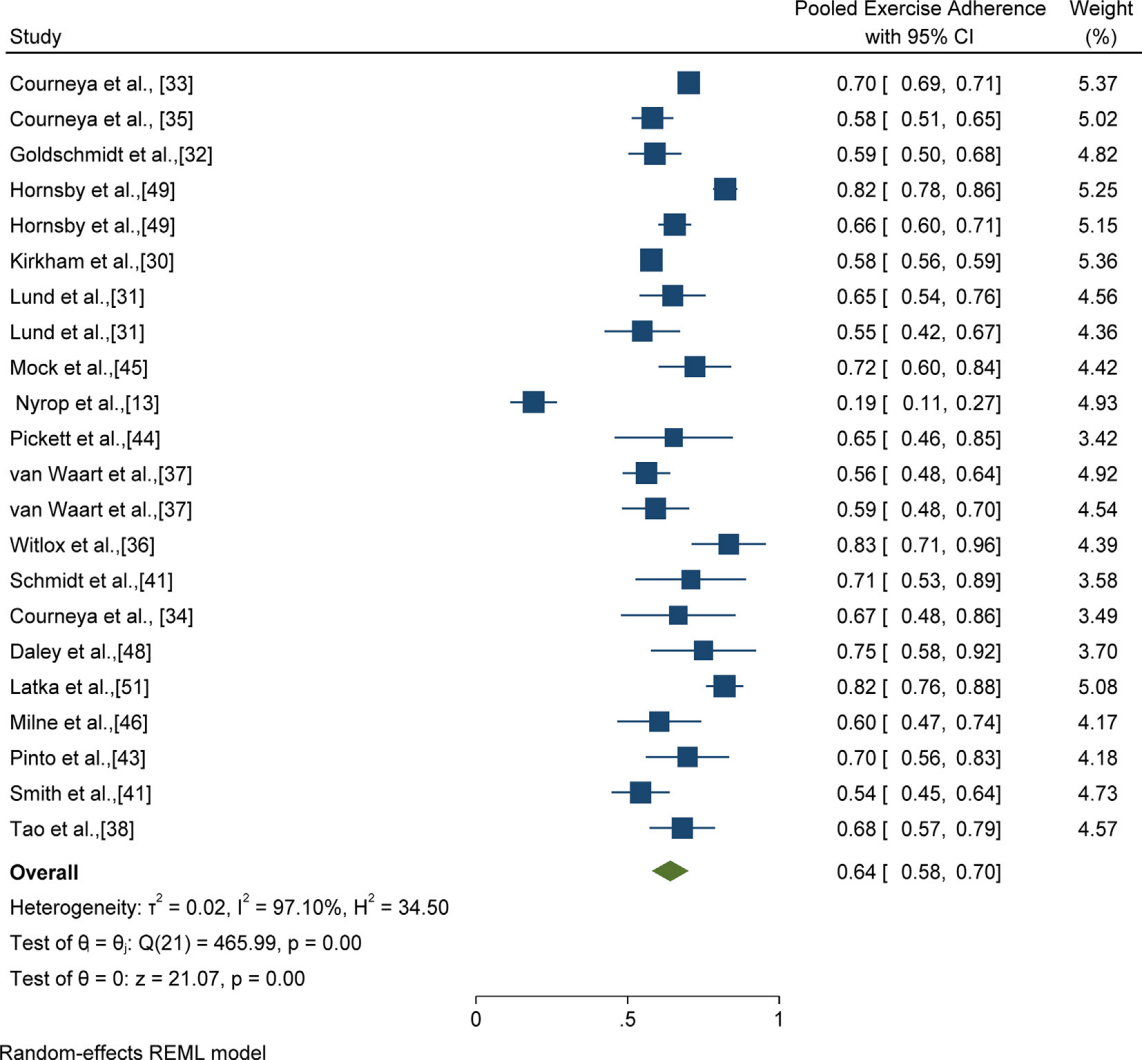
Pooled adherence to physical activity/exercise in persons with breast cancer.

Overall, twenty-six studies [13,16,[30], [31], [32], [33], [34], [35], [36], [37], [38], [39], [40], [41], [42], [43], [44], [45], [46], [47], [48], [49], [50], [51], [52], [53]] were included in the study. However, nineteen studies [13,[30], [31], [32], [33], [34], [35],^37^,^38^,[41], [42], [43], [44], [45], [46],^48^,^49^,^51^] provided sufficient data to be meta-analyzed. It is worth noting that the nineteen studies provided twenty-two exercise adherence values. This is because studies including Hornsby et al. [49], Lund et al. [31], and van Waart et al. [37] provided two adherence values each. The overall pooled exercise adherence of the nineteen studies with twenty-two adherence values was 64% [CI: 58%–70%]. However, the results were heterogeneous between studies [I^2^ ​= ​97.10%]. The results are presented in Fig. 2.

### 3.6 Sub-group analysis

Subgroup analysis was employed to investigate potential variations in estimates with respect to the stage of treatment, study design, environment, and the definition of adherence.

The pooled estimate for 16 studies during primary cancer treatment was 63% [CI: 55%–70%, I^2^ ​= ​98%], and for six studies after primary cancer treatment was 68% [CI: 59%–78%, I^2^ ​= ​73%]. However, there was no significant difference between the estimate before and after treatment [Q ​= ​0.82, p ​= ​0.36]. See Table 4.

**Table 4:** Sub-group analysis of possible moderating factors for exercise adherence.

Sub-group	k	Proportion [95%CI]	p-value	Test of group difference	I^2^ [p-value]
**Stage of treatment**					
During treatment	16	63% [55%–70%]	<0.001	Q ​= ​0.82, p ​= ​0.36	98.1% [<0.001]
After treatment	6	68% [59%–78%]	<0.001		73.4% [<0.001]
**Study design**					
Randomised controlled trial	17	68% [63%–73%]	<0.001	Q ​= ​2.59, p ​= ​0.11	87.7% [<0.001]
Observational	5	53% [35%–70%]	<0.001		96.5% [<0.001]
**Location**					
Centred based/supervised	13	67% [62%–72%]	<0.001	Q ​= ​1.03, p ​= ​0.31	95.1% [<0.001]
Home based	9	60% [48%–72%]	<0.001		92.9% [<0.001]
**Definition of adherence**					
Meeting exercise guidelines	4	66% [53%–79%]	<0.001	Q ​= ​2.51, p ​= ​0.21	89.3% [<0.001]
Attending session	6	70% [62%–79%]	<0.001		97.9% [<0.001]
Prescribed exercises	12	60% [51%–69%]	<0.001		90.0% [<0.001]
**Duration of interventions**					
≤12 weeks	9	69% [62%–75%]	<0.001	Q ​= ​0.87, p ​= ​0.35	86.7% [<0.001]
>12 weeks	8	61% [47%–75%]	<0.001		97.6% [<0.001]

I^2^ ​= ​Heterogeneity, k ​= ​number of studies.

Additionally, the pooled estimate for randomized controlled trials was 68% [CI: 63%–73%, I^2^ ​= ​88%] and for observational studies was 53% [CI: 35%–70%, I^2^ ​= ​96%]. See Table 4.

**Fig. 3:**
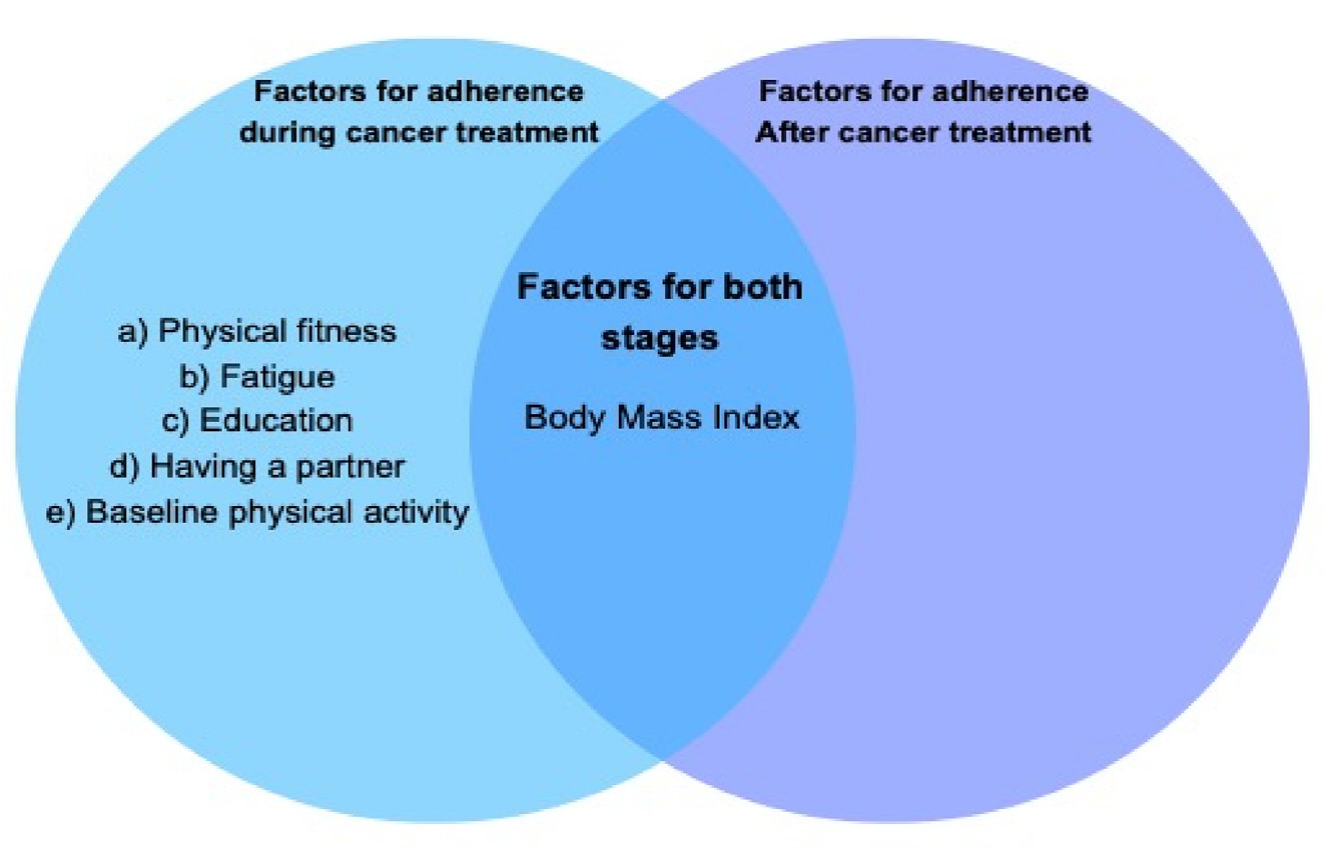
Potential factors influencing adherence to exercise during and after primary cancer treatment for women with breast cancer.

Moreover, the pooled estimate for center-based/supervised sessions was 67% [CI: 62%–72% I^2^ ​= ​95%], and for home-based adherence was 60% [CI: 48%–72%, I^2^ ​= ​93%]. See Fig. 3.

Furthermore, the pooled estimates based on different adherence definitions used in the various studies were 66% [CI: 53%–79%, I^2^ ​= ​89%], 70% [CI: 62%–79%, I^2^ ​= ​98%], 60% [CI: 51%–69%, I^2^ ​= ​90%] for meeting exercise/physical activity guideline, attending sessions, and adhering to prescribed exercise respectively. See Table 4.

Lastly, the pooled estimate for the duration of the intervention was 69% [62%–75%] for interventions lasting 12 weeks or less, and 61% [47%–75%] for interventions lasting more than 12 weeks. No significant difference was observed (Q ​= ​0.87, p ​= ​0.35). See Table 4.

### 3.7 Factors influencing adherence during and after primary breast cancer treatment

Five factors were identified to have significant influence on adherence to exercise during primary cancer treatment stage. Five studies [16,31,33,35,37] reported that physical fitness including muscle strength and aerobic capacity was positively associated with adherence to physical exercise. In three studies [35,36,52], fatigue was negatively associated with adherence to physical exercise. Additionally, three studies [32,37,40] reported that having a partner was positively associated with adherence to physical exercise. Three studies [13,35,39] reported that lower baseline physical activity levels were negatively associated with adherence to exercise and two studies [32,36] reported that education was positively associated with adherence to exercise in women with breast cancer.

Six studies [31,32,35,36,41,51] found that body mass index negatively correlated with adherence to physical exercise both after and during primary cancer treatment for women with breast cancer. The results are presented in Fig. 3.

### 3.8 Publication bias

**Fig. 4:**
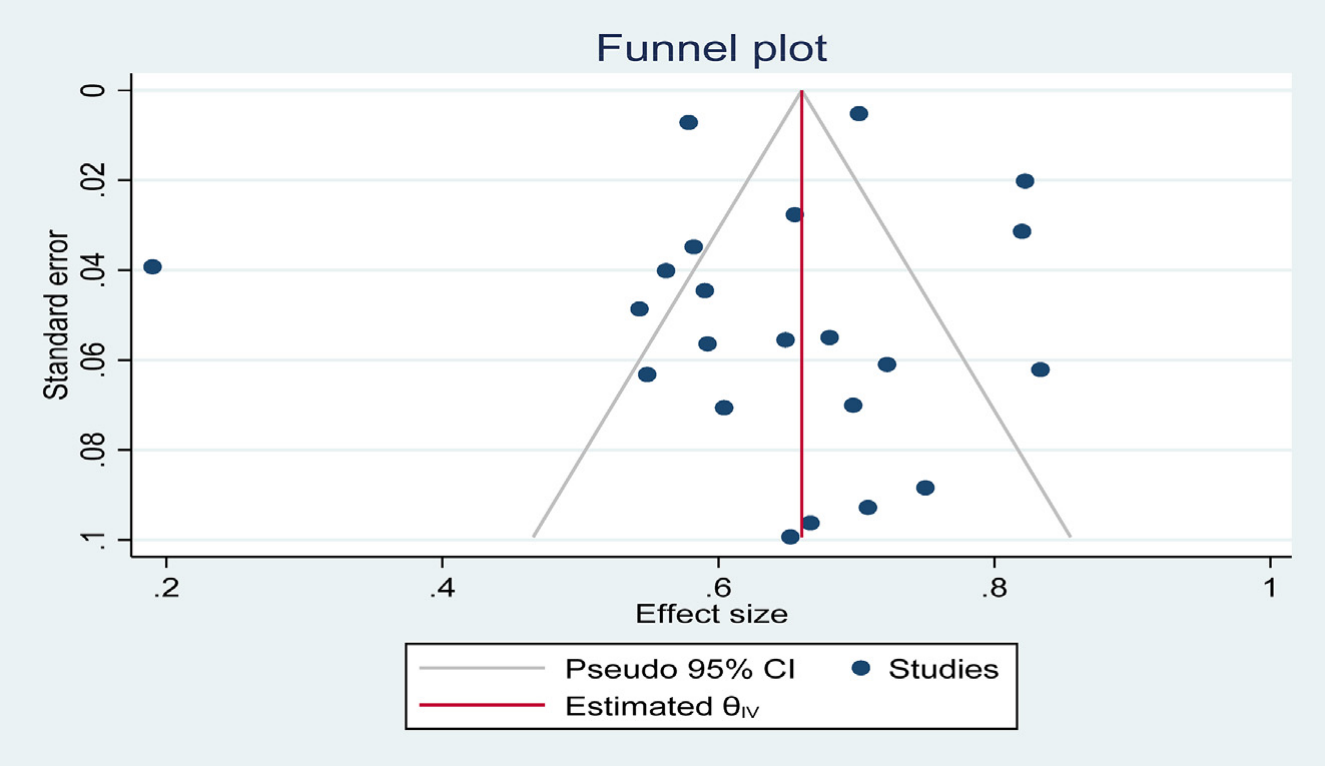
Assessing Publication bias by Funnel plot inspection.

No indication of publication bias was observed in either the subjective funnel plot [Fig. 4] or the objective Egger's regression test (z ​= ​0.37, p ​= ​0.7124).

## 4 Discussion

We conducted a systematic review and meta-analysis of adherence to exercise in breast cancer survivors, and to identify factors influencing adherence. Adherence was assessed either through meeting exercise and physical activity guidelines, attending exercise sessions, and adhering to prescribed exercises. We found that adherence to exercise was 64%. Specifically, adherence to exercise was comparatively similar in after primary cancer treatment (68%) and during primary cancer treatment (63%). Five factors including physical fitness, baseline physical activity, fatigue, education, and having a partner seemed to influence adherence during primary cancer treatments. Additionally, body mass index appeared to be associated with both stages of primary cancer treatments.

Contrary to our hypothesis, a non-significant difference was found in adherence to exercise among women with breast cancer between during and after primary cancer treatments. Additionally, there was potential overlap in their confidence interval estimates, and therefore, the difference might not be clinically significant either. It would have been expected that the potential number of barriers encountered during the treatment phase might moderate the adherence estimate, however, this was not the case. A possible contributing factor might be the health-seeking behaviors among the participants during and after their primary cancer treatment. Since breast cancer is a chronic condition [54], completing primary treatment does not necessarily mean a cure. As a result, individuals in both phases may equally continue to engage in healthy practices, such as adhering to exercise, physical activity, and diet, which could explain the absence of differences. Another plausible explanation could be that there were significant heterogeneities within each phase of treatment, suggesting that there could be unexplored factors, which might not have been included in the individual studies. Generally, measuring the actual adherence to exercise and physical activity in chronic conditions and the healthy population is challenging. This challenge could be attributed to the various ways adherence is measured in studies and practice, and therefore, the concept of adherence in rehabilitation is not well-established [55]. Taken together, more studies are warranted to explore adherence to exercise and potential contributing moderators among women with breast cancer during and after primary treatment.

Various factors were identified as potentially influencing adherence to exercise in the breast cancer population during cancer treatment, including physical fitness and baseline physical activity. Women with breast cancer who are physical fit and have higher baseline physical activity traits are more likely to adhere to exercise program. This could indicate that people within the breast cancer population who maintain a healthier lifestyle are more inclined to view exercise and physical activity as advantageous. Consequently, such people are more likely to actively engage in and strictly adhere to exercise intervention protocols. The perceived benefits of engaging in exercise and physical activity may serve as triggers for intrinsic motivation, a factor that is widely recognized for its association with persistent and committed participation in physical activities [56,57]. This further highlights that integrating healthy lifestyle including physical activity and fitness initiatives like ReSpAct [58,59] in daily life may have potential to increase future adherence to exercise program.

Fatigue was identified as a potential barrier to adherence to exercise/physical activity during primary cancer treatment. The association between fatigue and physical activity engagement is complex. While engagement in exercise may have the potential to reduce fatigue, individuals experiencing greater fatigue may perceive triggers for their symptoms during exercise participation [7,60]. Consequently, they are likely to drop out of exercise interventions. Regardless of the complex relationship, various studies and guidelines highlight the positive outcomes associated with the involvement of cancer and other populations in physical activity [[61], [62], [63], [64], [65]]. This implies that implementing strategies that facilitate the engagement and enjoyment of exercise without worsening fatigue among persons with breast cancer experiencing fatigue, such as balancing optimal rest and physical activity [66,67] as proposed for people with fatigue complaints, may show promise in exercise adherence and may be deemed advisable in this population.

Furthermore, having a partner was associated with adherence to exercise during primary breast cancer treatment. This suggests that having a partner may contribute to social and emotional support, thereby enabling engagement in exercise/physical activity programs during primary cancer treatment. The pivotal role of social support has been highlighted in previous studies on chronic conditions [68,69], also in the context of activity pacing [67,70]. Hence, when formulating exercise interventions and protocols for persons with breast cancer, it is advisable to incorporate considerations for this variable.

Six studies reported that body mass index may be negatively associated with exercise adherence in women with breast cancer during and after their primary treatments. This implies that women with breast cancer and lower body mass index were more likely to adhere to exercise interventions compared to their higher body mass index counterparts in both stages of primary cancer treatments. A previous review that focused on heterogenous cancer populations found inconsistent evidence for body mass index in the cancer population [19]. This implies that body mass index could be a unique factor for women with breast cancer and thus tailoring of interventions could be important. Furthermore, the consistent role of body mass index as a determinant of adherence across the phases of breast cancer treatment suggests that future interventions in exercise or physical activity for women with breast cancer should provide additional support for participants with higher body mass index.

### 4.1 Limitations and strengths

Our findings should be interpreted within the context of certain limitations. Notably, there was substantial heterogeneity observed in both the overall pooled adherence and sub-group analyses; thus, the interpretation of the findings should be considered carefully. The limited number of studies reporting on the stage of breast cancer and the type of exercise [aerobic and resistance training] during and after primary cancer treatment hampered further analysis on these domains.

Nevertheless, to our knowledge, this study represents the first systematic review and meta-analysis focusing on exercise adherence in a homogeneous breast cancer population. Furthermore, we identified differential factors influencing adherence for both before and after primary cancer treatment. The findings offer insights for tailoring exercise interventions to enhance participation in this specific population. The lack of evidence of publication bias in the included studies is a strength of the study. The study adherence to well-established systematic review and meta-analysis methodologies, aligning with internationally recognized standards and recommendations [23] provides validity for the reported findings.

## 5 Conclusion

The study demonstrated generally modest adherence and found no significant differences in exercise adherence among women with breast cancer during and after primary cancer treatment. Adherence was measured by meeting exercise/physical activity guidelines, attending exercise sessions, and adhering to prescribed exercises. Factors such as physical fitness, baseline physical activity, fatigue, education, and having a partner seemed to influence adherence during primary cancer treatments. Additionally, body mass index appeared to be associated with both stages of primary cancer treatments. Consequently, in the future development of interventions aimed at enhancing exercise participation among individuals with breast cancer, careful consideration of these identified factors is warranted to facilitate engagement in an active lifestyle.

## Confirmation of ethical compliance

Ethical approval is not required for this study.

## Funding and support

Ioulia Barakou is supported by the 10.13039/501100000269Economic and Social Research Council in United Kingdom funded NINE Doctoral Training Partnership (grant number: ES/P000762/1).

## Declaration of generative AI in scientific writing

During the preparation of this work, the author(s) used ChatGPT in order to improve the language and readability of the manuscript. After using this tool, the author(s) reviewed and edited the content as needed and take(s) full responsibility for the content of the publication.

## Declaration of competing interest

None.
